# MED12 Mutation in Two Families with X-Linked Ohdo Syndrome

**DOI:** 10.3390/genes12091328

**Published:** 2021-08-27

**Authors:** Luca Rocchetti, Eloisa Evangelista, Luigia De Falco, Giovanni Savarese, Pasquale Savarese, Raffaella Ruggiero, Luigi D’Amore, Alberto Sensi, Antonio Fico

**Affiliations:** 1 U.O. Genetica Medica della Romagna, Dipartimento di Patologia Clinica AUSL, 47522 Cesena, Italy; geneticamedica.ce@auslromagna.it (L.R.); asensi@ausl-cesena.emr.it (A.S.); 2 AMES-Centro Polidiagnostico Strumentale, Srl, 80013 Naples, Italy; eloeva@gmail.com (E.E.); genetica@centroames.it (G.S.); pasquale.savarese82@gmail.com (P.S.); raffaella.ruggiero@centroames.it (R.R.); ced@centroames.it (L.D.); centroames@libero.it (A.F.); 3 Fondazione Genetica per la Vita Onlus, 80013 Naples, Italy

**Keywords:** X-linked intellectual deficiency, next generation sequencing (NGS), *MED12*, genotype-phenotype correlation

## Abstract

X-linked intellectual deficiency (XLID) is a widely heterogeneous group of genetic disorders that involves more than 100 genes. The mediator of RNA polymerase II subunit 12 (MED12) is involved in the regulation of the majority of RNA polymerase II-dependent genes and has been shown to cause several forms of XLID, including Opitz-Kaveggia syndrome also known as FG syndrome (MIM #305450), Lujan-Fryns syndrome (MIM #309520) and the X-linked Ohdo syndrome (MIM #300895). Here, we report on two first cousins with X-linked Ohdo syndrome with a missense mutation in *MED12* gene, identified through whole exome sequencing. The probands had facial features typical of X-linked Ohdo syndrome, including blepharophimosis, ptosis, a round face with a characteristic nose and a narrow mouth. Nextera DNA Exome kit (Illumina Inc., San Diego, CA, USA) was used for exome capture. The variant identified was a c.887G > A substitution in exon 7 of the *MED12* gene leading to the substitution of a glutamine for a highly conserved arginine (p. Arg296Gln). Although the variant described has been previously reported in the literature, our study contributes to the expanding phenotypic spectrum of *MED12*-related disorders and above all, it demonstrates the phenotypic variability among different affected patients despite harboring identical mutations.

## 1. Introduction

X-linked intellectual deficiency (XLID) is a widely heterogeneous group of genetic disorders that involves more than one hundred genes [1]. MED12 is a member of Mediator complex (MED) that regulates gene expression in all eukaryotes and interacts with RNA polymerase II [2]. Mutations in MED12 have been shown to cause several forms of XLID, including Opitz-Kaveggia syndrome also known as FG syndrome (MIM #305450), Lujan-Fryns syndrome (MIM #309520) and the X-linked Ohdo syndrome (OSMKB subtype) (MIM #300895) [3,4,5,6,7,8,9,10]. These syndromes are allelic disorders that share clinical symptoms including ID, hypotonia and some physical features, such as tall prominent forehead, open mouth or high narrow palate.

To date, 77 MED12 variants have been described, in part due to the introduction of next generation sequencing techniques (NGS) [11]. Interestingly, only 8 of the 77 are associated with the XLID syndromes described above. Other variants have been described to cause syndromic, but not falling exactly in the three syndromes, or non-syndromic ID [12]. As is typically the case with X-linked related disorders, most patients with *MED12* mutations are male who have inherited the missense variant from the unaffected carrier mother [1]. Recently, however, females with *MED12* variants have been identified [2,3]. Furthermore missense, nonsense and frameshift variants were found.

In this report, we describe two first cousins, 7 and 8 years of age, presenting with mild to severe ID, speech delay, behavior problems and similar facial features, including blepharophimosis, ptosis, a round face with a characteristic nose and a narrow mouth. Using whole exome sequencing (WES), we discovered that both carry the *MED12* missense variant c.887G > A (p. Arg296Gln). This variant was first described by [4] in a male child that had features suggestive of OSMKB phenotype [4]. Further, in 2017, Patil et al. discovered the c.887 G > A mutation on two affected siblings with blepharophimosis-ID [5]. We finally compared clinical features among the two affected male cousins and other reported patients with *MED12* mutations/OSMKB phenotype.

## 2. Materials and Methods

### 2.1. DNA Extraction

Written informed consent for the study was obtained from the parents. Genomic DNA was extracted using peripheral blood in EDTA according to the manufacturer instructions (MagCore Nucleic Acid Extraction Kit Diatech Pharmacogenetics, Ancona, Italy).

### 2.2. Target Region Capture Sequencing and Bioinformatics Analysis

DNA quantification was performed using a Qubit 3.0 Fluorometer with the Qubit dsDNA HS (High Sensitivity) Assay Kit fluorescent dye method. Following the manufacturer instructions, we used 100 ng of DNA to perform clinical exome library preparation (Nextera DNA Exome kit, Illumina Inc., San Diego, CA, USA). After the target region sequence was captured and enriched, the resulting DNA libraries were quantified with the Qubit dsDNA HS Assay Kit fluorescent dye method to determine equimolar amounts for each library. Sequencing was carried out on NovaSeq 6000 (Illumina Inc., San Diego, CA, USA) to mean sequencing depth of at least 200×. Sequence data were aligned to the human reference genome GRCh37 (http://www.ncbi.nlm.nih.gov/projects/genome/assembly/grc/human/index.shtml (accessed on 11 October 2018)) using the Burrows-Wheeler Aligner with default parameters.

Trimming, base calling, coverage analysis, and variant calling were performed using an in house bioinformatic pipeline (bcl to fastq version 2.20, Isaac Aligner version 4, GATK “Genome Analysis Toolkit” version 4, Sam tools version 1.9 e Bed tools version 2).

Vcf analysis was performed using the Illumina Variant Interpreter filtered by quality > 15, by small variant consequences such as stop gains, sand losses, splice donors, splice acceptors, splice region, frameshift indels, in frame deletions, in frame insertions, initiator codon (ATG) losses, missense protein altering, incomplete terminal codon).Additional filtering was performed at frequency < 0.05 in European populations using tools such as the 1000 Genomes Project (https://www.internationalgenome.org/ (accessed on 11 October 2018)), gnomAD (https://gnomad.broadinstitute.org/), Exome Aggregation Consortium (http://exac.broadinstitute.org/ (accessed on 11 October 2018), and Human Gene Mutation Database (HGMD) (http://www.hgmd.cf.ac.uk/ac/ (accessed on 11 October 2018)). Variants were classified according to American College of Medical Genetics and Genomics (ACMG) guidelines [13]. We only selected variants affecting coding exons or canonical splice sites. Finally, synonymous variants were filtered out to detect only rare variants (frequency of <0.1%) in both dbSNP138 and our in-house database containing >1000 exomes) with high quality.

### 2.3. Sanger Sequencing Validation

Identified mutations were confirmed by Sanger sequencing in both the proband and the parents. Polymerase chain reaction (PCR) was performed to amplify the fragments of suspected candidate mutation loci, and Sanger DNA sequencing validation was performed to detect the corresponding loci in the probands and family members participating in the study. Primer Premier 5 software was used to design the target sequence primers (http://bioinfo.ut.ee/primer3-0.4.0/ (accessed on 11 October 2018)).

Primer sequences are available on request. The genomic sequence from GenBank accession number NM_005120.3 was used as reference sequence. PCR was performed in a 50 µL reaction volume to amplify the target fragments on a thermocycler instrument (PTC-200 PCR, BioRad, Milano, Italy), and the annealing temperature of PCR was 58 °C. The amplified products were isolated by electrophoresis on 1% agarose gel and purified using the QIamp purification kit (Qiagen, Valencia, CA, USA).

## 3. Results

### 3.1. Clinical Description

**Figure 1:**
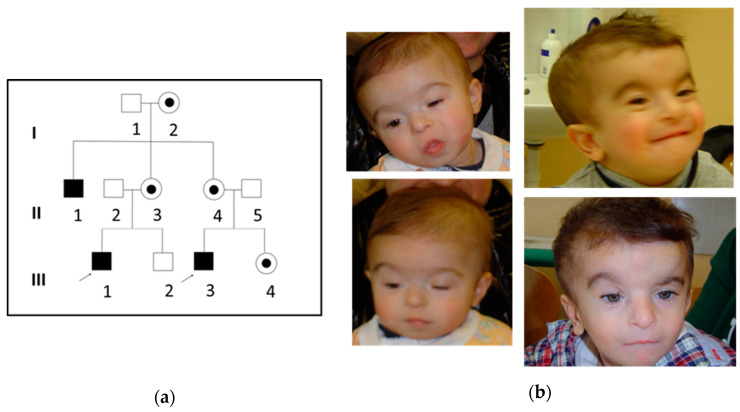
Pedigree and photographs. (**a**) Pedigree of the two probands. Filled circles represent carrier females, unfilled squares represent unaffected males, filled squares represent affected males. Arrow indicates the probands. (**b**) Facial dysmorphisms of the two affected subjects (III-1 on the left, III-3 on the right) with facial features consistent with X-linked Ohdo syndrome: thin and arched eyebrows, blepharophimosis, ptosis, round face with a characteristic nose and a narrow mouth.

We report on two affected males showing ID, developmental delay and facial dysmorphism who were referred to our hospital where a detailed clinical workup was performed (Figure 1). The males were first cousins; sons of two sisters who reported to have a brother with mild to severe ID and dysmorphic facial features. A DNA sample was obtained from the maternal uncle; however, detailed clinical information was unavailable. Clinical symptoms of the two probands are presented in Table 1. The parents reported that the two patients had a normal karyotype and were negative for fragile-X mutation.

#### 3.1.1. Patient 1

The proband III-1 is an 8-year-old male with ID, dysmorphic features and psychomotor delay. He was the first child of non-consanguineous healthy parents and presents harmonic short stature with neonatal development, height of 110 cm (3–10 percentile), and occipito-frontal circumference of 50 cm (3–10 percentile). On clinical examination he had sparse hair, arched and thin eyebrows, blepharophimosis, beaked nose, long and flat philtrum, cleft palate, micro-retrognathia and abnormal ears. Additional findings include up slanting palpebral fissures, sialorrhea and two inguinal hernias. At birth he showed clubfoot but this was corrected in early childhood with casts and stretching exercises. He had speech delay and language skills were limited to few words. The parents reported that he had a ventricular septal defect (VSD) and atrial septal defect (ASD) corrected by surgery.

#### 3.1.2. Patient 2

This 7-year-old male patient (III-3) showed multiple congenital anomalies, dysmorphic features and psychomotor delayed (Figure 1). The proband did not spontaneously walk and had difficulties performing routine daily living tasks. He was the first child of non-consanguineous healthy parents. The parents noted that he had tetralogy of Fallot corrected by surgery before the first year of life. Karyotype and array-CGH did not show any pathogenic chromosomal imbalance. Facial dysmorphic features include a long narrow face, a high forehead with frontal hair upsweep, a high nasal bridge and a long philtrum. He also showed arched and thin eyebrows, beaked nose, thick alae nasi, slight micro-retrognathia and sialorrhea, and brachycephaly. The eyelids are narrowed and he showed bilateral ptosis. He had inguinal hernia. On clinical examination he showed “café au lait” spots typically observed in incontinentia pigmenti but they were not distributed on Blaschko lines.

### 3.2. Molecular Findings

**Figure 2:**
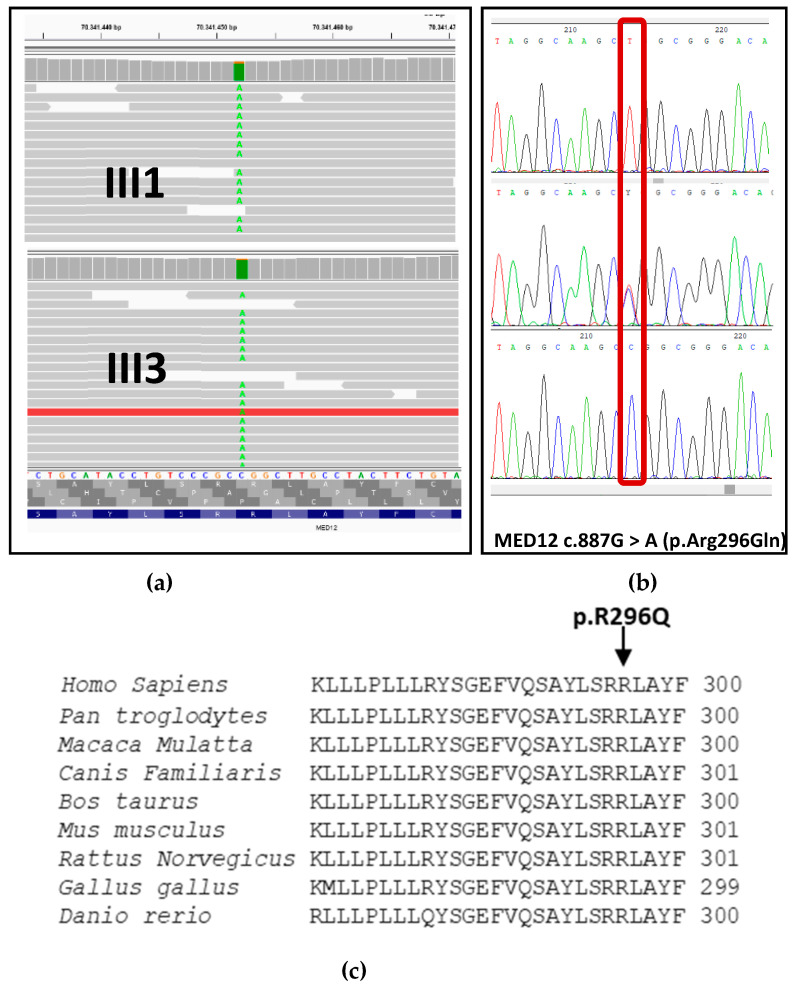
NGS data analyzed with IGV software and Sanger sequencing data analyzed with Chromas. (**a**) Photographs of IGV analysis reporting the c.887G > A, p.(R296Q) variant. Green line represents the variable base in IGV visualization (**b**) Sanger sequencing results of MED12 gene variant c.887G > A, p.(R296Q) in two affected individuals III-1, III-3 (hemizygous) and the unaffected mother II-3, II-4 (heterozygous). The site of the pathogenic variant is marked by a red line. (**c**) Alignment analysis of the amino acid sequences of MED12 from different species, showing complete conservation of the identified mutated residue (arrow).

Using WES, we identified a known missense variant in the MED12gene (Figure 2), which caused the substitution c. 887G > A in exon 7, and amino acid change arginine to glutamine at position 296 of the protein. Segregation analysis revealed a hemizygous variant in the affected boys and a carrier status in the unaffected mothers. Also, the mutation was confirmed in the maternal affected uncle. Sanger sequencing validated the identified pathogenic variant in all the affected subjects (Figure 2). The variant was predicted as damaging (Polyphen2, MutationTaster, SIFT) and it was absent in gnomAD database., The variant had been reported in ClinVar as likely pathogenic. Sequence alignment of the region of MED12 protein bearing the pathogenic variant Arg296Gln across closely related species revealed a high degree of conservation (Figure 2).

## 4. Discussion

Three different intellectual disability (ID) conditions that are associated with *MED12* mutations have been previously described. These conditions include Opitz- Kaveggia syndrome (FG syndrome type 1, FGS1), Lujan-Fryns syndrome (LFS), and X-linked Ohdo syndrome (XLOS, OHDOX) [1]. FG syndrome is characterized by ID, macrocephaly, broad thumbs, imperforated anus, corpus callosum hypoplasia or agenesis CCA and hypotonia. LFS is an X-linked intellectual disability syndrome associated with marfanoid habitus, macrocephaly, congenital hypotonia and hypernasal voice. OSMKB syndrome is characterized by ID, blepharophimosis, ptosis, long philtrum, micrognathia and hearing loss. Recently, different phenotypes that are not completely attributable to the known three MED12-related disorders, have been reported, thus expanding the spectrum of atypical phenotypes [6,7,8,9]. Females with varying degrees of cognitive impairment and different *MED12* mutations have been also reported [3,10]. In these individuals, nonsense and frameshift variants were identified in addition to the missense variants described in the well-known aforementioned conditions. The missense variants that cause disease in males and females were not clustered in one specific protein domain or motif and can cause different severity of (neuro)developmental disease in males and females [11]. In addition, truncating variants described in females and two males with divergent phenotypes are not related to a specific position in the MED12 protein. This suggests that it is not the position of the variant that determines phenotype, but rather the effect of the variant on MED12 activity [11].

Here, we presented two male probands exhibiting similar phenotypic characteristics of *MED12*-related disorders with developmental delay, ID, and dysmorphic features including tall prominent forehead, open mouth or high narrow palate. Moreover, our patients had speech delay, and facial features typically observed in Ohdo syndrome including blepharophimosis, ptosis, coarse face with a characteristic nose (thick alae nasi) and a small mouth. Neither corpus callosum dysgenesis which is a major criterion for LS/FGS nor congenital malformations such as imperforate anus that were commonly delineated in FGS were noticed in either patient. Exome sequencing performed in the two probands showed a missense mutation: c. 887G > A (p. Arg296Gln) which was confirmed by Sanger sequencing. The maternal uncle showing similar phenotypic features of the two probands was found to carry the same variant p. Arg296Gln. Segregation analysis of this mutation confirmed the X-linked transmission of the syndromic MED12 disorder (Figure 1).

The variant we described in the two affected subjects has been previously reported. The mutation was first described by Caro-Llopis in 2016 as a *de novo* MED12 mutation in a child that has features suggestive of OSKMB phenotype. The male patient presented with ID, Pierre-Robin sequence, blepharophimosis, microcephaly, chronic constipation and cryptorchidism [4]. In 2017, Patil et al. reported the c.887 G > A mutation on two affected siblings with blepharophimosis-ID [5]. The authors compared the clinical variability among the two affected male siblings and other reported patients with MED12 mutations/OSMKB phenotype [5]. The common clinical findings between our patients and those previously described include thick and arched eyebrows, blepharophimosis, small mouth, long and flat philtrum, micro-retrognathia, brachycephaly and abnormal ears. Additional clinical features shared by these probands include moderate-severe ID, psychomotor delay and a very limited use of expressive language.

Importantly, there are clinical features that do not match between the patients reported here and the patients reported by Caro-Llopis et al. and Patil et al. highlighting the interfamilial variability of identical *MED12* mutations. In particular, clinical findings not seen in our patients but reported in the patient described by Caro-Llopis et al. were severity of small mandible (requiring tracheostomy), bulbous nose, cleft palate, undescended testis, strabismus, chronic constipation and hypertrichosis [4]. Distinctive clinical features of the probands described by Patil et al. were unilateral high-placed winged scapula and left lower eyelid underdeveloped tarsal plate in the first proband, and hearing impairment and short stature in patient 2; features that have been reported in patients with OSMKB/MED12 mutation [5,8,12]. These differences suggest intrafamilial variability as they were not seen in our patients (excluding the bilateral ptosis present in one proband). In our patients, we observed additional clinical findings including sialorrhea, beaked nose and up-slanting palpebral fissures.

Congenital heart defect and genital abnormalities were clinical manifestations found in our patients and reported in one of the probands described by Patil et al. [5] and others [13,14] but not by Caro-Llopis et al. [4]. The proband described by Patil et al. showed peri-membranous ventricular septal defect (VSD) with posterior upper muscular extension and right coronary cusp (RCC) buckling at the age of 3 years [5]. Our patients (III-1, III-3) had congenital cardiac defects including ventricular septal defect and atrial septal defect in the first proband (III-1) and a tetralogy of Fallot in the second (III-2) [8,15], suggesting that congenital heart disease may be associated with mutations in the MED12 gene [6,16].

The clinical and phenotypic variability among the patients described here and by others is very wide [4,5]. One explanation for this could be due to how the MED12 protein functions in different pathways. MED12 is a part of a protein complex involved in the regulation of the majority of RNA polymerase II-dependent genes. It encodes a mediator of RNA polymerase II transcription subunit 12 [17]. MED12 gene mutations are known to alter cell-fate decisions and lead to a variety of pathologic conditions including developmental defects, cancers and heart development [18,19]. Very recently, the c. 887G > A, (p.Arg296Gln) missense variant observed in our patients was described in two Italian cases who presented with recurrent conotruncal heart defect, common arterial trunk (CAT) and aorto-pulmonary window (APW), respectively and facial dysmorphisms [20]. In this paper, Amodeo et al. focused mainly on the cardiac malformations rather than on facial dysmorphisms, probably because the molecular investigations were performed during the prenatal period when the clinical phenotype was not well defined [20]. Nevertheless, these findings confirmed that the same genes can be involved in different pathways and this could explain the pleiotropic effects on different organs of *MED12* [21]. Furthermore, rather than the mutations affecting the same protein interactions within the mediator complex, perhaps the mutations, affect other domains implicated in distinct protein interactions and these may result different clinical consequences. Although the variant we describe here has already been reported, this study contributes to the growing phenotypic spectrum of *MED12*-related disorders and above all, it highlights the clinical variability of the same mutation among different affected subjects.

## 5. Conclusions

In this paper, we confirmed the involvement of the MED12 gene in ID. Although the number of MED12 mutations reported to date is too limited to define genotype–phenotype correlations, mutations in different protein regions might cause variability in clinical consequences including more severe cognitive impairment or more pronounced dysmorphic features. Our results expand the genotype and phenotype spectrum of MED12-mediated XLID syndromes and allow for a deeper understanding of its molecular pathogenesis.

## Figures and Tables

**Table 1:** Comparison of clinical findings among the three well-known MED12-related syndromes (FG, Lujan-Fryns and Odho), the probands with Arg296Gln variation previously described [4,5] and the two probands with Arg296Gln mutation described in the present paper.

Feature Category	Feature	FG Syndrome/ Opitz-Kaveggia Arg961Trp, Gly958Glu	Lujan-Fryns Syndrome Asn1007Ser	Odho Syndrome Arg1148His, Ser1165Pro, His1729Asn	[4] Arg296Gln	[5] Arg296Gln	III-1 Arg296Gln	III-3 Arg296Gln
General	ID	+	+	+	+	+	+	+
	Developmental delay	+	+	+	+	-	-	-
	Poor/absent speech	-	-	-	+	-	+	+
	Hypernasal speech	-	+	-	-	-	-	-
	Tall stature	-	+	-	-	-	-	-
	Thin habitus	-	+	+	+	-	+	+
Central nervous system	Neonatalhypotonia	+	+	+	-	+	-	-
	Corpus callosumagenesis (CAA)	+	+	-	-	-	-	-
	Seizures and EEG abnormalities	+		+	-	-	-	-
	Spasticity with joint contracture	+	-	-	-	-	-	-
Craniofacial	Macrocephaly	+	+	-	-	-	-	-
	Microcephaly/brachycephaly	-	-	+	+	+	+	+
	Long narrow face	+	+	-	-	-	-	-
	Triangular face	-	-	+	-	+	+	+
	Coarse face	-	-	+	-	-	-	-
	Prominent forehead	+	+	+	-	+	+	+
	Sparse eyebrows	-	+	+	-	-	-	-
	Thick and arched eyebrows	-	-	-	+	+	+	+
	Hypertelorism	+	-	+	+	+	-	-
	Thickalae nasi	-	-	+	-	-	+	+
	Low set ears	-	-	-	+	+	+	+
	Narrow lips	-	-	+	-	-	+	+
	High, arched palate	+	+	+	-	-	-	-
	High nasal root	+	+	-	-	-	-	-
	Long philtrum	-	+	-	+	+	+	+
	Cleft Lip/Palate	-	-	+	-	-	-	-
	Dental abnormalities	-	+	+	-	+	-	-
	Maxillary hypoplasia	+	-	+	-	-	-	-
	Prominent nasal bridge	-	+	-	-	+	-	-
	Micrognathia	+	+	+	+	+	+	+
	Blepharophimosis	-	-	+	+	+	+	+
	Ptosis	-	+	+	-	+	+	+
	Down-slanted palpebral fissures	+	+	+	-	+	-	-
	Up-slanted palpebral fissures	-	-	-	-	-	+	+
Ophthalmologic	Strabismus	+	+	+	+	-	-	-
	Nystagmus	+	-	-	+	-	-	-
Auditory	Hearing Loss	-	-	+	-	-	-	-
Musculoskeletal	Skeletal anomalies	+	-	+	+	-	-	-
	Broad thumbs and halluces	+	+	-	-	-	-	-
	Long super-extendible digits	-	+	-	-	-	-	-
Cardiopulmonary	Cardiac abnormalities	+	+	+	-	+	+	+
Gastrointestinal	Gastrointestinal anomalies	+	-	+	-	-	-	-
	Chronic constipation	+	-	+	+	-	-	-
Genitourinary	Cryptorchidism	+	-	+	+		-	-
	Inguinal hernia	+	-	-	-	+	+	+
Behavior	Characteristic behavior	+	+	+	-	+	-	-

## Data Availability

Not applicable.

## References

[1] Rubinato E., Rondeau S., Giuliano F., Kossorotoff M., Parodi M., Gherbi S., Steffan J., Jonard L., Marlin S. (2020). MED12 Missense Mutation in a Three-Generation Family. Clinical Characterization of MED12-Related Disorders and Literature Review. Eur. J. Med Genet..

[2] Li D., Strong A., Shen K.M., Cassiman D., Van Dyck M., Linhares N.D., Valadares E.R., Wang T., Pena S.D.J., Jaeken J. (2021). De Novo Loss-of-Function Variants in X-Linked MED12 Are Associated with Hardikar Syndrome in Females. Genet. Med..

[3] Polla D.L., Bhoj E.J., Verheij J.B.G.M., Wassink-Ruiter J.S.K., Reis A., Deshpande C., Gregor A., Hill-Karfe K., Silfhout A.T.V., Pfundt R. (2021). De Novo Variants in MED12 Cause X-Linked Syndromic Neurodevelopmental Disorders in 18 Females. Genet. Med..

[4] Caro-Llopis A., Rosello M., Orellana C., Oltra S., Monfort S., Mayo S., Martinez F. (2016). De Novo Mutations in Genes of Mediator Complex Causing Syndromic Intellectual Disability: Mediatorpathy or Transcriptomopathy?. Pediatr. Res..

[5] Patil S., Somashekar P., Shukla A., Siddaiah S., Bhat V., Girisha K., Rao P. (2017). Clinical Variability in Familial X-Linked Ohdo Syndrome–Maat-Kievit-Brunner Type with MED12 Mutation. J. Pediatr. Genet..

[6] Lesca G., Moizard M.-P., Bussy G., Boggio D., Hu H., Haas S.A., Ropers H.-H., Kalscheuer V.M., Des Portes V., Labalme A. (2013). Clinical and Neurocognitive Characterization of a Family with a Novel *MED12* Gene Frameshift Mutation. Am. J. Med. Genet..

[7] Bouazzi H., Lesca G., Trujillo C., Alwasiyah M.K., Munnich A. (2015). Nonsyndromic X-linked Intellectual Deficiency in Three Brothers with a Novel *MED12* Missense Mutation [c.5922G>T (p.Glu1974His)]. Clin Case Rep..

[8] Langley K.G., Brown J., Gerber R.J., Fox J., Friez M.J., Lyons M., Schrier Vergano S.A. (2015). Beyond Ohdo Syndrome: A Familial Missense Mutation Broadens the *MED12* Spectrum. Am. J. Med. Genet..

[9] Tzschach A., Grasshoff U., Beck-Woedl S., Dufke C., Bauer C., Kehrer M., Evers C., Moog U., Oehl-Jaschkowitz B., Di Donato N. (2015). Next-Generation Sequencing in X-Linked Intellectual Disability. Eur. J. Hum. Genet..

[10] Charzewska A., Maiwald R., Kahrizi K., Oehl-Jaschkowitz B., Dufke A., Lemke J.R., Enders H., Najmabadi H., Tzschach A., Hachmann W. (2018). The Power of the Mediator Complex-Expanding the Genetic Architecture and Phenotypic Spectrum of *MED12* -Related Disorders. Clin. Genet..

[11] van de Plassche S.R., de Brouwer A.P.M. (2021). MED12-Related (Neuro)Developmental Disorders: A Question of Causality. Genes.

[12] Maat-Kievit A., Brunner H.G., Maaswinkel-Mooij P. (1993). Two Additional Cases of the Ohdo Blepharophimosis Syndrome. Am. J. Med. Genet..

[13] Isidor B., Lefebvre T., Le Vaillant C., Caillaud G., Faivre L., Jossic F., Joubert M., Winer N., Le Caignec C., Borck G. (2014). Blepharophimosis, Short Humeri, Developmental Delay and Hirschsprung Disease: Expanding the Phenotypic Spectrum of *MED12* Mutations. Am. J. Med. Genet..

[14] Vulto-van Silfhout A.T., de Vries B.B.A., van Bon B.W.M., Hoischen A., Ruiterkamp-Versteeg M., Gilissen C., Gao F., van Zwam M., Harteveld C.L., van Essen A.J. (2013). Mutations in MED12 Cause X-Linked Ohdo Syndrome. Am. J. Hum. Genet..

[15] Verloes A., Bremond-Gignac D., Isidor B., David A., Baumann C., Leroy M.-A., Stevens R., Gillerot Y., Héron D., Héron B. (2006). Blepharophimosis-Mental Retardation (BMR) Syndromes: A Proposed Clinical Classification of the so-Called Ohdo Syndrome, and Delineation of Two New BMR Syndromes, One X-Linked and One Autosomal Recessive. Am. J. Med. Genet..

[16] Napoli C., Schiano C., Soricelli A. (2019). Increasing Evidence of Pathogenic Role of the Mediator (MED) Complex in the Development of Cardiovascular Diseases. Biochimie.

[17] Wang H., Shen Q., Ye L., Ye J. (2013). MED12 Mutations in Human Diseases. Protein Cell.

[18] Hong S.-K., Haldin C.E., Lawson N.D., Weinstein B.M., Dawid I.B., Hukriede N.A. (2005). The Zebrafish Kohtalo/Trap230 Gene Is Required for the Development of the Brain, Neural Crest, and Pronephric Kidney. Proc. Natl. Acad. Sci. USA.

[19] Segert J., Schneider I., Berger I.M., Rottbauer W., Just S. (2018). Mediator Complex Subunit Med12 Regulates Cardiac Jelly Development and AV Valve Formation in Zebrafish. Prog. Biophys. Mol. Biol..

[20] Amodeo S., Vitrano G., Guardino M., Paci G., Corselli F., Antona V., Barrano G., Magliozzi M., Novelli A., Venezia R. (2020). What Is the Impact of a Novel MED12 Variant on Syndromic Conotruncal Heart Defects? Analysis of Case Report on Two Male Sibs. Ital. J. Pediatr.

[21] Rocha P.P., Scholze M., Bleiß W., Schrewe H. (2010). Med12 Is Essential for Early Mouse Development and for Canonical Wnt and Wnt/PCP Signaling. Development.

